# Macrofungi as a Nutraceutical Source: Promising Bioactive Compounds and Market Value

**DOI:** 10.3390/jof7050397

**Published:** 2021-05-19

**Authors:** Allen Grace Niego, Sylvie Rapior, Naritsada Thongklang, Olivier Raspé, Wuttichai Jaidee, Saisamorn Lumyong, Kevin D. Hyde

**Affiliations:** 1 Center of Excellence in Fungal Research, Mae Fah Luang University, Chiang Rai 57100, Thailand; agniego27@gmail.com (A.G.N.); naritsada.t@gmail.com (N.T.); ojmraspe@gmail.com (O.R.); 2 School of Science, Mae Fah Luang University, Chiang Rai 57100, Thailand; 3 Iloilo Science and Technology University, La Paz, Iloilo 5000, Philippines; 4 Laboratory of Botany, Phytochemistry and Mycology, Faculty of Pharmacy, CEFE, CNRS, University Montpellier, EPHE, IRD, CS 14491, 15 Avenue Charles Flahault, CEDEX 5, 34093 Montpellier, France; sylvie.rapior@umontpellier.fr; 5 Medicinal Plants Innovation Center, Mae Fah Luang University, Chiang Rai 57100, Thailand; wuttichai.jai@mfu.ac.th; 6 Department of Biology, Faculty of Science, Chiang Mai University, Chiang Mai 50200, Thailand; scboi009@gmail.com; 7 Research Center of Microbial Diversity and Sustainable Utilization, Chiang Mai University, Chiang Mai 50200, Thailand; 8 Academy of Science, The Royal Society of Thailand, Bangkok 10300, Thailand; 9 Innovative Institute of Plant Health, Zhongkai University of Agriculture and Engineering, Guangzhou 510408, China

**Keywords:** bioactivities, macrofungi, medicinal properties, market value, nutraceuticals, nutrients

## Abstract

Macrofungi production and economic value have been increasing globally. The demand for macrofungi has expanded rapidly owing to their popularity among consumers, pleasant taste, and unique flavors. The presence of high quality proteins, polysaccharides, unsaturated fatty acids, minerals, triterpene sterols, and secondary metabolites makes macrofungi an important commodity. Macrofungi are well known for their ability to protect from or cure various health problems, such as immunodeficiency, cancer, inflammation, hypertension, hyperlipidemia, hypercholesterolemia, and obesity. Many studies have demonstrated their medicinal properties, supported by both in vivo and in vitro experimental studies, as well as clinical trials. Numerous bioactive compounds isolated from mushrooms, such as polysaccharides, proteins, fats, phenolic compounds, and vitamins, possess strong bioactivities. Consequently, they can be considered as an important source of nutraceuticals. Numerous edible mushrooms have been studied for their bioactivities, but only a few species have made it to the market. Many species remain to be explored. The converging trends and popularity of eastern herbal medicines, natural/organic food product preference, gut-healthy products, and positive outlook towards sports nutrition are supporting the growth in the medicinal mushroom market. The consumption of medicinal mushrooms as functional food or dietary supplement is expected to markedly increase in the future. The global medicinal mushroom market size is projected to increase by USD 13.88 billion from 2018 to 2022. The global market values of promising bioactive compounds, such as lentinan and lovastatin, are also expected to rise. With such a market growth, mushroom nutraceuticals hold to be very promising in the years to come.

## 1. Introduction

Macrofungi (from the Greek “makros”, meaning large), includes members of the phylum Basidiomycota, and a few Ascomycota that fruit above or below the ground, with large sporocarps or fruiting bodies that can be seen with the unaided eye [1]. Macrofungi, collectively referred to as mushrooms, are distributed throughout the world, with about 14,000 species globally [2]. About 350 species of mushrooms are consumed around the world [3]. The most cultivated edible mushrooms worldwide are *Agaricus bisporus* (button mushroom), *Flammulina velutipes* (enoki mushroom), *Lentinula edodes* (shiitake mushroom), and *Pleurotus* spp., in particular oyster mushroom [4,5,6,7]. In the last decade, China has made some significant breakthroughs in the breeding and cultivation techniques of edible mushrooms, as well as product innovations, which has led to increased mushroom production [8]. Many people consume mushrooms because of health-promoting benefits [9], a driving force in the increased market value of medicinal mushrooms [10]. At least 270 species of macrofungi have been explored as sources of important secondary metabolites and have the potential to be developed as food supplements for medicinal applications [11].

Macrofungi production and economic value have increased globally [2]. Because of the increasing mushroom demand, it is expected that the total value of the global mushroom market per annum will exceed USD 50 billion in the coming few years [12]. The global market value of edible mushrooms is forecasted to reach up to USD 62.19 billion in 2023 [13]. The popularity of macrofungi among consumers has expanded, owing to their pleasant and unique flavors, as well as for their health benefits [14]. Since the 20th century, the demand for food has increased and the requirements for consumables have become more stringent [15]. Food should not only provide nourishment and satisfy hunger, but should also aid in the improvement of the physical and psychological condition of humans and especially aid in preventing and treating diseases [16]. Many bioactivities of macrofungi, such as anticancer, antidiabetics, antihypertensive, antimicrobial, anti-inflammatory, antioxidant, immunomodulatory, cholesterol lowering, neurotrophic, and neuroprotective properties, have been well studied [17,18,19,20]. Macrofungi, both wild and cultivated, can be seen as healthy functional food [17]. The fresh fruiting bodies of macrofungi have a moisture content of approximately 70–95%. The dry matter is composed of carbohydrates (50–65%), proteins (19–35%), and essential fatty acids (2–6%), with traces of vitamins and minerals [21]. Edible mushrooms could be a source of many different nutraceuticals, such as β-glucans, lectins, unsaturated fatty acids, phenolic compounds, tocopherols, ascorbic acid, and carotenoids. Thus, consumption of edible macrofungi promotes health, taking advantage of the additive and synergistic effects of all the bioactive compounds present [22]. Hence, macrofungi are potentially important sources of low-calorie functional foods and nutraceuticals [2].

The most widely cultivated edible macrofungi are *A. bisporus* (button macrofungi), *Auricularia auricula* (wood ear macrofungi), *F. velutipes* (winter macrofungi), *L. edodes* (shiitake), *Pleurotus* spp. (oyster macrofungi), and *Volvariella volvacea* [23,24]. Moreover, macrofungi such as *Ganoderma lingzhi–G. sichuanense* (Lingzhi or Reishi), *Inonotus obliquus* (Chaga), *L. edodes* (Shiitake), and many others have been collected in the wild and utilized as medicines for hundreds of years in many Asian countries, such as Korea, China, Japan, and eastern Russia. Such practices have prompted modern scientific studies of fungal medicinal properties, especially anticancer bioactivities [25]. The most explored species for their medicinal value are *Antrodia cinnamomea*, *Ganoderma lingzhi–G. sichuanense, Ophiocordyceps sinensis, Phellinus linteus,* and *Xylaria nigripes* [17,26,27]. Numerous bioactive compounds have been found in their fruiting bodies or cultured mycelium, such as alkaloids, carotenoids, enzymes, fats, folates, glycosides, lectins, minerals, organic acids, phenolics, polysaccharides, proteins, terpenoids, tocopherols, and volatile compounds in general [5,28,29]. The fruiting bodies of macrofungi have approximately 70–95% moisture content, with abundant carbohydrates (50–65%), proteins (19–35%), and some fats (2–6%) [21], in which many are bioactive constituents, such as polysaccharides, biologically active proteins (enzymes, lectins, and ergothioneine), unsaturated fatty acids (oleic and linoleic), phenolic compounds (phenolic acids and polyphenols), vitamins (A, B complex, C), and dietary fibers [26,30].

This review aims to establish the potential of mushrooms as nutraceuticals, with emphasis on their medicinal properties and bioactive compounds. Experimental in vivo and in vitro studies, and clinical trials supporting the bioactivities of the different compounds from edible mushrooms are compiled. The market value of medicinal mushrooms and bioactive compounds from market studies is also established in this review. Macrofungal resources have not been fully explored yet, thus a lot of studies are still needed to unlock their diverse potential applications [2].

## 2. Nutritional Value of Mushrooms

The moisture content of fresh mushrooms varies from 70 to 95%, depending on the environmental conditions and time of harvest. Mushrooms are known to be rich in high quality protein, and to contain a high proportion of unsaturated fatty acids as a form of vegetable. Mushrooms are also a good source of fiber, particularly the soluble fiber β-glucan [31]. The fruiting bodies of mushrooms are made up of 50 to 60% of carbohydrates on a dry weight basis, with free sugars amounting to 11% [32]. The carbohydrate content of *Pleurotus ostreatus* (white strain) was reported to be 56% [33]. The most dominant mushroom sugar is mannitol which constitutes about 80% of the total free sugars [34]. Proteins are an important functional component of mushroom fruiting bodies. Mushrooms, in general, have a higher protein content than most other vegetables and most wild plants. Based on the data published by the USDA in 2019, 100 g of raw mushrooms contains only 22 kcal. Overall, nutrients are present as follows: carbohydrates (3.26 g/100 g DW), protein (3.09 g/100 g DW), fiber (1.0 g/100 g DW), and fat (0.34 g/100 g DW). Mushrooms have a low glycemic index and are presumed to have little negative effect on blood glucose and insulin response [35]. Mushrooms are also full of micronutrients, such as the vitamin B complex, including vitamin B5, which assists in the release of energy from carbohydrates, proteins, and fat [29]. Mushrooms also contain a high level of mineral elements that are essential for human health. Major mineral constituents in mushrooms are K, P, Na, Ca, and Mg, as well as minor elements, including Cu, Zn, Fe, Mo, and Cd [36]. The content in minerals depends mostly on the substrates supplied for mushroom cultivation, as well as the species, age, and size of the fruiting bodies. Wild mushrooms tend to have higher mineral contents compared to cultivated ones [37]. Table 1 lists some edible mushrooms and their moisture, protein, ash, fat, and carbohydrate contents.

## 3. Nutraceuticals

Nutraceuticals are a group of products that are more than food but less than pharmaceuticals, in that they can be considered a supplement to effective pharmacological treatment. There is no internationally accepted definition yet, thus the term nutraceuticals can have various meanings depending on the country [48]. The word “nutraceuticals” was originally coined in 1989 by Stephen De Felice, founder and chairman of the Foundation for Innovation in Medicine [49]. The term nutraceutical refers to a product that must have a beneficial effect on health, proven by clinical testing [50]. Nutraceuticals provide medical or health benefits for the prevention and treatment of disease [51], thus playing a vital role in human health and longevity [52]. They can be delivered to the consumer as a dietary supplement and/or as a functional food. The nutraceutical industry encompasses three main segments, which include functional foods, dietary supplements, and herbal/natural products. Nutraceuticals are not proposed as an alternative to drugs, but can be helpful to complement a pharmacological therapy and help in preventing the onset of chronic diseases in subjects who do not qualify for conventional pharmacological treatment [50]. Many clinical studies have been carried out to support the effectiveness, as well as the general safety of many nutraceuticals. However, since nutraceuticals can be used even without the approval of authorities, there are certain risks associated with their consumption, such as the possibility of dangerous interactions with medications, especially in vulnerable populations [48].

Although around 270 species of mushrooms have medicinal properties [11], only a handful are considered as nutraceuticals. Species that are most commonly found in dietary supplements include: *A. bisporus* (button mushroom), *O. sinensis* (cordyceps), *G. lingzhi* (Reishi), *Grifola frondosa* (maitake), *Hericium erinaceus* (lion’s mane), *L. edodes* (shiitake), and *Trametes versicolor* (turkey tail) [5,53]. Many edible mushrooms may still be classified as nutraceuticals in the near future. Such future discoveries are likely since many mushroom species that have been utilized traditionally by different cultures throughout the world for prevention and treatment of many diseases remain to be scientifically studied. “Mushroom nutraceuticals” [54] was coined since mushrooms were used even in ancient times in the form of extracts, health tonics, concentrates, fermented beverages, tinctures, teas, soups, herbal formulas, powders, and healthy food dishes. In addition to nutritional contents, many studies have documented the bioactivities of mushrooms with pharmaceutical potential in the last decades, describing mushrooms as mini pharmaceutical factories of bioactive compounds [55]. These remarkable discoveries have drawn attention outside of the scientific community to the use of mushrooms as bioactive ingredients in functional food products that can increase the nutritional qualities of these products. Examples of food considered to potentially benefit from macrofungi include: bread, muffins, pasta, patties, and snacks [56,57,58]. Other processed food products have also been incorporated with mushrooms, thus increasing the popularity of mushrooms among consumers. As a consequence, the awareness of the healthful benefits of incorporating mushrooms in the diet has increased. Currently, around 5 kg of mushrooms are consumed per person per year, and this is expected to increase in the years to come [53].

Species that produce secondary metabolites that have a wide range of biological activities are considered as medicinal macrofungi [59]. Many studies have documented the medicinal properties of mushrooms, including antitumor [60], immunomodulating, antioxidant [61], radical scavenging, cardiovascular-protective [62], antihypercholesterolemia, antiviral, antibacterial, antiparasitic, antifungal, detoxifying, hepatoprotective, and antidiabetic effects [63]. Many polysaccharides or polysaccharide–protein complexes exhibit antitumor activities in animals and humans by enhancing innate and cell-mediated immune response [60].

## 4. Bioactive Compounds from Macrofungi and Their Medicinal Properties

### 4.1. Mushroom Species Containing Bioactive Polysaccharides

Polysaccharides are the most potent substance derived from mushrooms and are responsible for various physiological activities, like antitumor, immunomodulatory, antioxidant, antiviral, anti-inflammatory, anticarcinogenic, and neuroprotective activities [19,22]. Many studies have documented that some sugars produced by mushrooms, such as rhamnose, xylose, fucose, arabinose, fructose, glucose, mannose, mannitol, sucrose, maltose, and trehalose, possess bioactivities [5]. Biologically active polysaccharides (glucans derivatives) produced from macrofungi exhibit various structures with different properties [60]. The antitumor activity of polysaccharides depends primarily on their chemical structures. Many glycans, which are homopolymers to highly complex heteropolymers, exhibit antitumor activities. These compounds activate the immune response of the host organisms. Thus, the antitumor activity is indirectly targeting the tumor cells. The compounds prevent stress in the body, which helps in the reduction of tumor cells and increased survival rate of tumor-induced mice [64,65]. Table 2 lists some important macrofungi and the bioactivities of polysaccharides from experimental studies.

*Agaricus bisporus* polysaccharides have antiaging activity, and can protect hepatic and renal functions by improving serum enzyme activities, biochemical levels, lipid contents, and antioxidant status [66,115,116]. ABP-1 and ABP-2 fractions of polysaccharides demonstrated antitumor activity by inhibiting the growth of human breast cancer MCF-7 cells and reducing the growth of murine sarcoma 180 cells implanted subcutaneously into mice [117]. AlAPS and AcAPS and their major purified fractions have antiaging and antioxidant effects. Specifically, AcAPS-2 showed scavenging activity on hydroxyl (82.98 ± 4.67%) and DPPH (64.47 ± 4.05%) radicals at the concentration of 1.0 mg/mL in vitro [68]. Both AlAPS and AcAPS demonstrated hepatic and nephric protection activities by improving serum enzyme activity, biochemical levels, lipid contents, and antioxidant status [66,68]. ABP and fractions Abnp1002 and Abap1002 also demonstrated hepato-protective activity against CCl_4_-induced hepatic injury in mice [7,69]. The mannogalatoglucan extracted and characterized from *A. bisporus* showed antitumor activity against human hepatocarcinoma cells (HepG2) by inducing apoptosis via the mitochondrial death pathway [67]. ABP also showed immunostimulatory effects in RAW 264.7 cells [70].

*Ganoderma* spp. polysaccharides are also very promising nutraceuticals with multiple bioactivities, such as anti-angiogenesis, antidiabetic, antioxidation, antiproliferation, hepatoprotection, and immunomodulation [118]. GLP has been shown to prevent glioma growth in glioma-bearing rats by increasing the concentration of serum interleukin-2, tumor necrosis factor-α, and interferon-γ, as well as enhance the cytotoxic activity of natural killer cells and T cells [75]. It also exerts antitumor activity via MAPK pathways in HL-60 acute leukemia cells [119]. GLP also inhibited the proliferation of colorectal cancer HT29 (p53R273H) and SW480 (p53R273H&P309S) cells using the p53-mediated tumor-suppressing pathways [72]. Furthermore, GLP suppressed the growth and migration of LNCaP human prostate cancer cells [74]. GLP also inhibited the accumulation of myeloid-derived suppressor cells (MDSC) via the CARD9-NF-κB-IDO pathway, thus preventing lung cancer development [120]. Polysaccharides from *G. lingzhi/G. sichuanense* (as *G. lucidum*) were also shown to promote cognitive function and neural progenitor proliferation in a mouse model of Alzheimer’s disease [121] and had a neuroprotective effect by regulating the expression of apoptosis-associated proteins, inhibiting oxidative stress-induced neuronal apoptosis [73]. GLPs also had hypoglycemic effects in diabetic mouse models (T2DM), in which glucose levels and lipid metabolism were improved [76,78]. However, degraded polysaccharide, GLP_UD_, was proven to have stronger hypolipidemic and antioxidant activities than GLP in mice, which could significantly improve lipid metabolism disorders in hepatocytes [77].

*Grifola frondosa* polysaccharides are also very promising antitumor agents. In fact, a *G. frondosa* polysaccharide-based drug was developed in China and approved as an adjunctive therapeutic drug for cancer treatment [122]. The cancer-fighting ability of GFP was superior to polysaccharides from *G. sichuanense*, lentinan, and *Trametes versicolor* [122]. Many studies have demonstrated the inhibitory effects of polysaccharides from *G. frondosa* mycelial extracts on different cancer-cell-bearing mice. GFP inhibited the growth of LoVo and HT-29 human colon cell proliferation and induced cytotoxicity [113]. It also exerted cytotoxic effects on the MCF-7 and MDA-MB-231 cells, as well as in nude mice bearing MCF-7 tumor xenografts, as indicated by a decrease in cell viability, increase in the apoptotic rate, and induced mitochondrial dysfunction supporting its potential application to cure breast cancer [79]. Aside from anticancer activity, GFP also showed hypoglycemic and hypolipidemic activities. A study in diabetic mice induced by HFD and STZ administered with GFP showed reduced sugar and lipid levels in the blood by altering gut microbiota [82]. *Grifola frondosa* polysaccharide-N (GFP-N) also demonstrated hypoglycemic effects in diabetic mouse livers [81]. GFP administration also significantly improved memory impairment in aged rats via antioxidant action, thus can be a good dietary supplement for brain aging [80].

Similar to other macrofungi discussed so far, *Hericium erinaceus* polysaccharides (HEP) also possess various bioactivities. They have been extensively studied for potential and existing applications in pharmaceuticals and functional foods [29]. The different polysaccharides isolated from this species are galactoxyloglucans, glucoxylans, hetero- xyloglucans, and xylans [123]. *Hericium erinaceus* exhibit antitumor, immunomodulatory, antioxidative, gastroprotective, neuroprotective, neuroregenerative, hypolipidemic, hypoglycemic, antifatigue and antiaging properties [124]. Bioactive compounds extracted from the mycelia and fruiting bodies of *H. erinaceus* have been found to promote nerve growth factors accompanying cell proliferation [125]. They improve recognition memory [126] and have been used to treat cognitive impairment, Parkinson’s disease, and Alzheimer’s disease by promoting the expression of neurotrophic factors that are associated with cell proliferation, such as nerve growth factors [125]. The enzymatic modification of HEP (EHEP) significantly enhanced phagocytosis of NO, CD40, and CD86 positive cells by peritoneal macrophages, thus enhancing the immunomodulation function of HEP [88]. The hydroxyethylated derivative of HEP also has potential immunomodulatory activities on RAW264.7 peritoneal macrophages [83]. Moreover, sHEPs also showed strong immunostimulant activity by inducing dendritic cell maturation through MAPK and NF-κB signaling pathways [84]. Pretreatment of HEP also reduced ethanol-induced gastric mucosal lesions and pylorus ligation-induced gastric ulcers in mice, which is further evidence for its gastroprotective activity [85]. A novel polysaccharide from *H. erinaceus* (HEP_N_) also exhibited gastroprotective activity by preventing H_2_O_2_-induced oxidative stress from damaging human gastric epithelium (GES-1) cells by promoting cell proliferation, inhibiting cell necrosis, reducing ROS levels, regulating mitochondrial membrane potential, and maintaining mitochondrial membrane permeability [86]. The novel polysaccharide HEFP-2b also inhibited the growth of colon cancer cells (HCT-116) in vitro by arresting it in the S-phase of the cell cycle [87].

*Lentinula edodes* polysaccharides show antitumor activity against human cervical carcinoma HeLa cells, causing an inhibitory effect on their proliferation and induced apoptotic death [90]. They also inhibit the proliferation of HT-29 colon cancer cells and suppress the growth of tumors in athymic nude mice [127]. LEP impairs the immunosuppressive function of myeloid-derived suppressor cells via the p38 pathways [89]. The mannogalactoglucan-type polysaccharides (WPLE-N-2 and WPLE-A0.5-2) from. *L. edodes* exhibit anticancer and immunomodulating activities on sarcoma 180 (S-180)-bearing mice [64]. LEPs are also known for their immunomodulatory effects. The three fractions of LEP (F1, F2, and F3) demonstrated immunomodulatory effects through enhancing cellular immunity by increasing the thymus index, DTH, and proliferation of T splenocytes [93]. RPS and ERPS of *L. edodes* also exhibit antioxidative, anti-inflammatory, and organ protective effects against the LPS-induced sepsis in mice, which makes them suitable for functional foods and treatment of sepsis and its complications [91]. The acidic spent mushroom compost polysaccharides (ASMCP) also have antioxidant, anti-inflammatory, and renoprotective effects against LPS-induced KI in mice [92].

*Ophiocordyceps sinensis* polysaccharides are also a very promising source of bioactive polysaccharides with anticancer, antihypertensive, anti-inflammatory, anti-obesity, and antioxidant activities, as well as drug delivery capacity, and can also help as a prebiotic. The docetaxel-loaded acetic acid conjugated *O. sinensis* polysaccharide (DTX-AA-CSP) was demonstrated to have antitumor activity in vivo against human liver (HepG2) and colon cancer cells (SW480), which was even more effective than the currently marketed treatment of docetaxel injection (Taxotere^®^) [99]. CSP also significantly inhibited the proliferation of human colon cancer cell line HCT116 cells, resulting in increased autophagy and apoptosis [100]. CPS-A also showed a good protective effect on angiotensin (Ang II)-induced L02 cell injury [95]. CSP1-2 fraction also demonstrated an antihypertensive effect on spontaneously hypertensive rats (SHR) by stimulating the secretion of vasodilator NO, decreasing the level of ET-1, epinephrine, noradrenaline, and angiotensin II, inhibiting the increase of transforming growth factor β1 (TGF-β1) and lowering the level of inflammatory mediator of C-reactive protein (CRP) [94]. CSP soluble dietary fiber had protective effects against obesity on HFD-feeding C57BL/6J mice; however, the study also showed that, though CSP could prevent the increase in body weight, it could also result in aggravated liver fibrosis and steatosis [98]. CSP has also been shown to improve microbial community diversity and modulated the overall structure of gut microbiota, thus can be a potential prebiotic agent [96,97].

*Pleurotus eryngii* polysaccharides inhibit the growth of human hepatoblastoma HepG-2 cells [101]. PEP isolated and purified also possessed good immunoregulatory activity in vivo, and stimulated the production of NO and cytokines by MAPK and NF-κΒ [103]. PEP extracted with hot water exhibited hypolipidemic and hypoglycemic activities, measured by decreased body weight gain, levels of plasma insulin, serum triglyceride, low-density lipoprotein cholesterol, and blasting blood glucose in mice, and thus could be explored as a possible therapeutic agent for hyperlipidemia and hyperglycemia [102]. High-dose PEP treatment also had lipid-lowering and liver protection effects on mice with hyperlipidemia [128]. PEP also significantly elevated cell viability, reduced the levels of intracellular calcium, and decreased β-amyloid-mediated cell apoptosis in PC12 cells of mice, thus represents a possible therapeutic approach to ameliorate the onset and progression of Alzheimer’s disease [105].

*Pleurotus ostreatus* polysaccharides have more diverse bioactivities. Numerous animal studies with *P. ostreatus* documented the hypoglycemic, hypolipidemic, and antioxidant effects [108]. These polysaccharides can also reduce hyperglycemia and hyperlipidemia levels in STZ-induced diabetic rats by improving insulin resistance and increasing glycogen storage [129]. POP also regulated dyslipidemia of hyperlipidemia rats [108], thus lowering the chance of having premature atherosclerosis, which could lead to angina or heart attack. Moreover, POP can alleviate cognitive impairment in rats, suggesting that these polysaccharides can help cure Alzheimer’s disease [106]. Consumption of *P. ostreatus* may improve glucose and lipid metabolism, blood pressure, body weight, and appetite sensations [130]. PPOP exhibited stronger hepatoprotective effects and stronger antioxidant activity in vivo when compared to unphosphorylated POP [111]. Different studies also demonstrated the antitumor and anticancer activities of POP against targeted cells. POP has cytotoxic activity when applied to murine lymphoid cancer cell line [109] and sarcoma 180 tumor cells [107]. A novel selenium polysaccharide fraction (Se-POP-3) can also can induce apoptosis and inhibit migration of cancer cells [110].

*Trametes versicolor* polysaccharides also showed numerous bioactivities, especially against different cancer types. The direct toxicity of TVP preparations to cancer/tumor cells has been demonstrated in the various in vitro models, as discussed by Habtemariam [131]. Numerous cancer types were targeted by the polysaccharides isolated from *T. versicolor*. TVP inhibits human colon cell proliferation and induces cytotoxicity [113]. TVP also had antihyperlipidemic effects in HFD-induced hyperlipidemic mice, improving serum lipid profiles [114]. It can also improve HaCaT cell survival, owing to its antioxidant property [112].

### 4.2. Macrofungal β-Glucans

**Figure 1:**
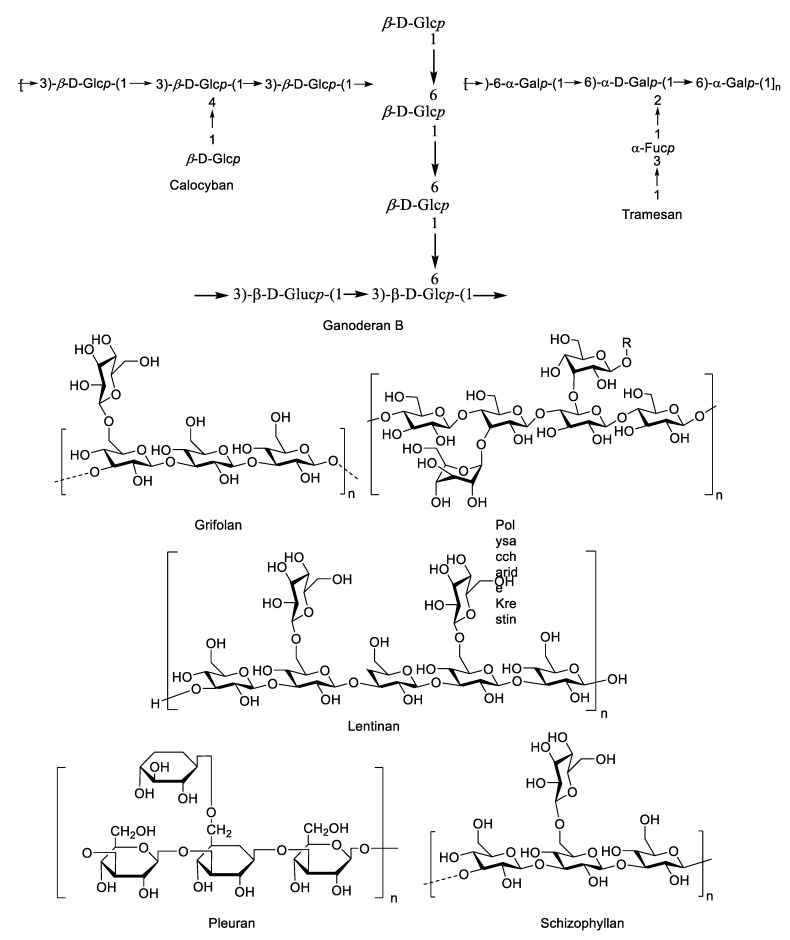
Chemical structures of *β*-glucans isolated from some economically important macrofungi.

Among the different polysaccharides, β-glucans are the most abundant in mushrooms, and are found primarily in the fungal cell wall. They are the most versatile metabolite, with a wide spectrum of biological activity [132]. Detailed studies of β-glucans demonstrated their beneficial impact on human life. These compounds are responsible for many bioactivities, such as immunomodulating, anticholesterolemic, antidiabetic, antioxidant, and neuroprotective activities, as well as lipid balance improvement, and they even have a great impact on the general feeling of the consumer [133]. They bind to a membrane receptor and induce these biological responses [134]. Since β-glucans are not synthesized by the human body, they therefore induce both innate and adaptive immune responses [135]. Chemical structures of important β-glucans can be found in Figure 1.

Calocyban is a polysaccharide isolated from *Calocybe indica*. Mandal et al. [136] isolated new water-soluble (1→6)-, (1→4)-α, β-glucan and water-insoluble (1→3)-, (1→4)-β-glucan (calocyban) from alkaline extracts of this mushroom. Extracts of this macrofungus also showed synergistic effects with standard antibiotics. It increased the efficacy of ciprofloxacin against opportunistic pathogenic bacteria [137]. The ethanolic extract of *C. indica* also exhibited an antiproliferative and apoptotic effect on PANC-1 and MIAPaCa2 cell lines of pancreatic cancer in vitro [138].

Ganoderan is a hypoglycemic polysaccharide derived from the aqueous extract of *G. lingzhi/G. sichuanense* (as *G. lucidum*) [139]. The ganoderans A, B, and C were all isolated from this species and demonstrated bioactivity against non-small-cell lung carcinoma (NSCLC), and had the effects of hyperglycemic and kidney protection [140,141]. Many studies have been documented on the bioactivities of ganoderans. The study of Wang et al. [140] showed that ganoderan B can be used to inhibit growth of H510A and A549 cells by suppressing the expression of ki67 and proliferating cell nuclear antigen (PCNA), thus it can be effective in suppressing NSCLC tumor formation and metastasis. Ganoderan B promotes apoptosis of H510A and A549 cells by regulating the expression of Bcl-2, Bax, cleaved caspase 3, and cleaved poly (adenosine diphosphate-ribose) polymerase (PARP). It also demonstrated hypoglycemic activity by increasing the plasma insulin level in normal and glucose-loaded mice [142]. Moreover, the ganoderans obtained from the mycelial fractionation of *G. lingzhi/G. sichuanense* (as *G. lucidum*) IY009 also exhibit immunomodulating effects. The ganoderan isolated from the cell wall of *G. lingzhi/G. sichuanense* IY009 showed the highest antitumor activity (inhibition rate of 94%) in sarcoma-bearing mice and 37% of anticomplementary activity [143].

Grifolan is the polysaccharide, branched (1,3)-β-glucan, extracted from the fruit body or the mycelium of the fungus *G. frondosa* [144]. In the earlier study of Takeyama et al. [145], grifolan NMF-5N was shown to have a host-mediated antitumor effect. Although grifolan NMF-5N did not exhibit a direct effect on the tumor cells, intraperitoneal injection of this compound increased the number of peritoneal exudate cells and peritoneal adherent cells. The increase in number of these cells indicated cytostatic activity towards syngeneic tumor cells. Grifolan as an immune-modulator was also confirmed by Ishibashi et al. [146], where it activated macrophages to produce tumor necrosis factor (TNF) in vitro. β-glucan, isolated by Seo et al. [147] also showed immunostimulatory activity, confirmed through cell activation ability and cytokine expression. In accordance with the molecular evidence suggesting the polysaccharides from this fungus may have health benefits, in Asia, *G. frondosa* or maitake are consumed and recommended to treat various diseases, such as arthritis, hepatitis, and HIV [148].

Polysaccharide Krestin (PSK) is a β-glucan–protein complex consisting of 25–38% protein residues, with a molecular weight of about 94 KDa. It is isolated from *T. versicolor*, demonstrating strong antitumor activity against numerous cancer types and has been used as an adjuvant for cancer treatment with no known side effects [149]. This complex mainly consists of acidic amino acids, such as aspartic acid and glutamic acid, neutral amino acids, such as valine and leucine, and small amounts of basic amino acids, such as lysine and arginine. In the early studies, PSK was shown to have direct effects on the gene expression profile in cancer cells which inhibit hepatic carcinogenesis in rats induced by 3′-methyl-4-dimethylaminoazobenzene [150,151]. PSK has long been used in Asia, and recently in Western countries, as a treatment for cancer due to its presumed immune potentiating effects [152]. Coriolan as a β-(1→3) polysaccharide with some (1→6) and no (1→4) branched glucan, also isolated from *T. versicolor*, showed to be effective (100 mg/kg for 30 days) in suppressing sarcoma 180 tumors in mice [153].

Lentinan is a β-glucan isolated from *L. edodes*. The structure of lentinan is composed of a β-(1–3)-glucose backbone with two (1–6)-β-glucose branches of each five glucose units [154]. Many clinical studies have verified the efficacy of lentinan to treat various cancers, such as colorectal cancer, gastric cancer, lung cancer, and ovarian cancer. Lentinan demonstrated antioxidative, antitumor (fibrosarcoma), and antimetastatic activities [154,155,156,157], immune potentiating activity [158,159], anti-inflammatory [156], and even antimicrobial activity [160].

Since 1985, lentinan has been used in Japan as an adjuvant for stomach cancer therapy [161]. A case of recurrent ovarian cancer successfully treated with adoptive immunotherapy and lentinan has been reported by Fujimoto et al. [162]. In general, the indirect anticancer, as well as immunostimulatory effects of lentinan have been attributed to the activation of many immune cells [163]. Lentinan can increase the engulfing ability of certain immune cells to search and destroy migratory cancer cells in the human body [164]. Specifically, lentinan can activate immunocytes (NK, macrophage, T cells) by triggering MAPK-NF-κB and Syk-PKC into binding with recognition receptors, such as TLR2/4/6/9, Dectin-1, and other membrane receptors [157]. Lentinan also activates the NRF2-ARE signaling pathway to prevent cis-DDP-induced kidney injury in vivo [165].

Lentinan also has antibacterial activity against *Mycobacterium tuberculosis* [166] and *Listeria monocytogenes* [167]. This β-glucan also helps in improving the bactericidal ability of peritoneal and alveolar macrophages [168]. It has immunomodulating effects against the Newcastle disease virus [169] and malarial infection [170], thus can be developed into antiviral and antimalarial drugs. Lentinan based drugs can also be used for treating HIV [171,172], hepatitis, and malignant pleural effusion [154]. Studies on chemotherapy combined with lentinan exhibited better efficacy and response rates than chemotherapy alone in treating different kinds of cancer [157]. The adverse effects of chemotherapy, such as leukopenia, thrombocytopenia, vomiting, etc., were lessened with the supplementation of lentinan, showing a significant improvement in the quality of life and physical condition for patients with breast, colorectal, gastric, gynecological, hepatic, intestinal, and lung cancers, and lymphoma [157]. Clinical data compiled in the past 12 years and presented in the review by Zhang et al. [158] showed that lentinan is effective not only in improving quality of life, but also in promoting the efficacy of chemotherapy during lung cancer treatment.

Pleuran, the β-glucan (β-(1,3/1,6)-D-glucan) produced by the *P. ostreatus*, has also demonstrated biological response modifier properties. It has proven to be an effective supplement to increase the immunity of athletes, even for intensive training [173]. This β-glucan was found to be effective against upper respiratory tract infections [174,175]. Pleuran has also been shown to have antiviral activity against herpes simplex virus type 1 (HSV-1). Another study [176] showed promising results in the clinical and immunomodulatory effects of pleuran-based supplements against HSV-1. Active treatment with pleuran in systemic application caused a significantly shorter duration of herpes simplex symptoms compared to a placebo group. Moreover, pleuran demonstrated effectiveness against acute respiratory symptoms, with the duration and severity of respiratory symptoms lower in the pleuran applied group compared to the placebo group.

Schizophyllan is a nonionic, water-soluble homoglucan, neutral extracellular polysaccharide, β-(1-6)-branched β-(1-3)-glucan, produced by the fungus *Schizophyllum commune* [177]. It has numerous uses for commercial, nutraceutical, and medicinal applications. Schizophyllan is quite similar to lentinan in its composition, biological activity, and mechanism of immunomodulation and antitumor action [177]. This homoglucan exhibits bioactivities, such as acting as a biological response modifier and nonspecific immuno-stimulator [178]. The antitumor activity of schizophyllan is mainly due to the host-mediated immune response, enhancement of cell-mediated immune response with stimulation of T lymphocytes and macrophages, and improving cytokine production [177,178]. It can counter various diseases and help to enhance the effects of vaccines and antitumor therapies [179]. Other reported bioactivities include antineoplastic, antibacterial, and antiparasitic properties [164].

Tramesan is an exopolysaccharide released in the culture filtrate of *T. versicolor*, which acts as an antioxidant regardless of the biological system to which it is applied [180]. Tramesan can decrease immune system depression, act as an antioxidant, prevent cancer, inhibit growth of *Candida albicans*, along with having antiviral activity by inhibiting the replication of HIV, and further possessing liver protective functions [181]. It has also been found to induce a marked growth inhibition of leukemic cell lines and primary cells from acute myeloid leukemic patients [182]. Additionally, Tramesan was also found to have agricultural applications. It can be used as a natural alternative to crop protection chemicals for controlling the mycotoxins produced by *Aspergillus flavus* and *A. carbonarius* [183]. Finally, this exopolysaccharide inhibits the growth of wheat pathogens, *Zymoseptoria tritici* and *Parastagonospora nodorum* [184].

### 4.3. Proteins

Proteins from macrofungi have many pharmaceutical activities and possess immunomodulatory properties, and antitumor, antiviral, antibacterial, and antifungal activities. Bioactive proteins have great value in terms of pharmaceutical potential. Examples of such proteins include lectins, fungal immunomodulatory proteins, ribosome inactivating proteins, antimicrobial proteins, and ribonucleases [185].

Lectins are carbohydrate binding proteins that can aggregate immunoglobulins and may be involved in sugar transport or carbohydrate storage in the cell [186]. They are present in the mushroom fruiting bodies with important roles in the life cycle of these fungi [187]. Lectins manifest diverse bioactivities, including antitumor, immunomodulatory, antifungal, HIV-1 reverse transcriptase inhibitory, and anti-insect activities [188]. Moreover, they can also inhibit fungal growth and induce the release of histamines [186]. Some proteins exhibit highly potent antiproliferative and anti-metastasis activity toward some tumor cell lines (human leukemic T cells, hepatoma Hep G2 cells, and breast cancer MCF7 cells) [185]. A lectin isolated from *Ganoderma applanatum* was shown to have antiproliferative activity in HT-29 colon cancer cells [189]. The in vivo immunomodulatory effects of lectin isolated from *A. bisporus* was also studied [190]. Lectin from *P. ostreatus* (POL) stimulated an immune response and has been considered as a potential therapeutic approach to break HBV tolerance [191]. It has also been shown to act as a food-intake-suppressing substance, which can help in weight reduction [192]. A lectin-like protein of unknown function designated as a light subunit of mushroom *A. bisporus* tyrosinase (LSMT) showed inhibition of cell growth of breast cancer cells and light stimulation of cell proliferation of macrophage cells. These homologous proteins display the ability to penetrate the intestinal epithelial cell monolayer, and are adequate for oral administration. Just like other lectins it can be developed as a drug carrier and anticancer treatment [193].

**Figure 2:**
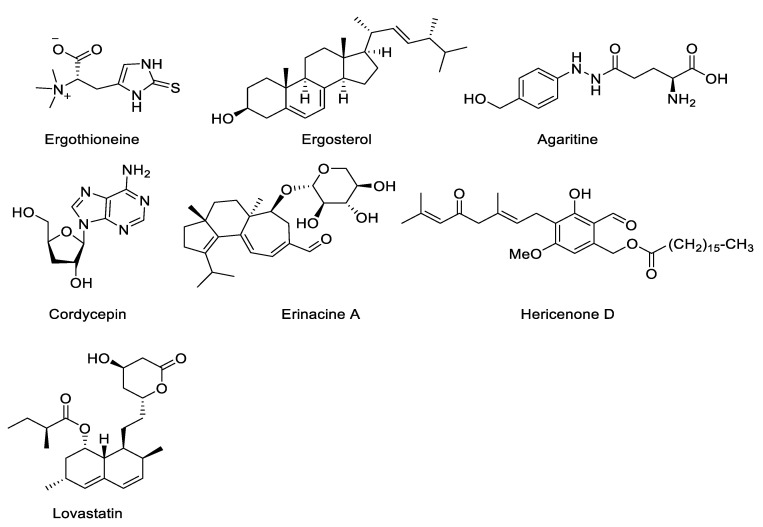
Chemical structures of other bioactive compounds found in macrofungi.

Ergothioneine is an amino acid that is found in some mushrooms, such as *A. bisporus* [194] and *Pleurotus citrinopileatus* [195] (Figure 2). It is a thiourea derivative of histidine, containing a sulfur atom bonded to the 2-position on the imidazole ring [196]. Early studies showed that humans are unable to synthesize this compound, and its presence in the blood is mostly dependent on diet [194]. Ergothioneine demonstrated antioxidant and cytoprotective capacities in vitro [194,196,197]. These functions may highlight therapeutic benefits against numerous conditions in humans. Cheah & Halliwell [198] proposed that ergothioneine could be used as a therapeutic to reduce the severity and mortality of COVID-19.

Flammulin is a ribosome-inactivating protein (RIP) from the fruiting bodies of *F. velutipes*. It has a molecular weight of 40 kDa, and is capable of inhibiting cell-free translation in a rabbit reticulocyte lysate system, with an IC_50_ of 0.25 nM [199]. Flammulin is an antitumor substance [200], with inhibitory effects on the proliferation of sarcoma 180 and Ehrlich ascites tumors [201,202]. This protein also affected immune reactions and had positive effects on the therapy of various types of cancer. Hemagglutinin is another promising bioactive compound isolated from the fruiting bodies of *F. velutipes*. It can stimulate [3H-methyl] thymidine uptake by mouse splenocytes and inhibits proliferation of leukemia L1210 cells [203].

### 4.4. Fats

The amount of fats in mushroom fruiting bodies is low compared to carbohydrates and proteins. The fats present in mushrooms are mostly unsaturated fatty acids. Mushrooms are rich in linolenic acid, which is an essential fatty acid [204]. Other lipids, such as tocopherols, are essential fatty acids that take part in a wide range of physiological functions. They have high antioxidant activities which help in protecting the body against degenerative malfunctions, cancer, and cardiovascular diseases [5,204,205]. Some lipid components, including fatty acids, fatty acid esters, and sterols, were identified from the ethyl acetate extract of *Cordyceps militaris*. This extract at 10 μg/mL concentration was able to reduce the NO production in Bv2 cells by 85% via activation of NRF2 and NF-κB transcription. Moreover, it downregulated inflammatory genes, iNOS and COX-2, and upregulated anti-inflammatory genes, HO-1 and NQO-1 [206], which could be very useful in treating neurodegenerative diseases [207]. The lipids extracted from the ethyl acetate fraction of *Pleurotus giganteus* also showed antifungal activity by significantly inhibiting the growth of all the *Candida* species tested in the study by Phan et al. [208].

Ergosterol, also produced in mushrooms, has been shown to have antioxidant properties [209], which helps prevent cardiovascular diseases [210] (Figure 2). Ergosterol (ergosta-5,7,22-trien-3β-ol) is a sterol found in cell membranes of fungi and protozoa that plays an important role in fundamental biological processes, such as signal transduction, cellular sorting, cytoskeleton reorganization, asymmetric growth, and the response to infectious diseases [211]. This compound is necessary for the survival of many fungi and protozoa. The enzymes that synthesize ergosterol have become important targets for antifungal drug discovery [212]. Ergosterol is a steroid precursor of vitamin D2. In human nutrition, exposure to small amounts of ultraviolet light is required for the activation of vitamin D. Among the cultivated mushrooms, the *Pleurotus* species have been shown to have relatively higher concentrations of ergosterol, with better conversion to vitamin D2 [213,214]. Extracts from organically produced *L. edodes* also contain the high content of ergosterol [215]. Agarol is an ergosterol derived from *Agaricus blazei* which has anticancer properties, inhibiting the proliferation of A549, MKN45, HSC-3, and HSC-4 human carcinoma cell lines [216].

### 4.5. Phenolic Compounds

Phenolic compounds are secondary metabolites possessing an aromatic ring with one or more hydroxyl groups, and their structure can be that of a simple phenolic molecule or a complex polymer. Phenolic compounds in mushrooms are excellent antioxidants and synergists, while not being mutagenic. They also exhibit a wide range of physiological properties, such as antiallergenic, antiatherogenic, anti-inflammatory, antimicrobial, antithrombotic, cardioprotective, and vasodilator effects [5]. Phenolic compounds are well documented for their antioxidant activity as free radical scavengers, singlet oxygen quenchers, or metal ion chelators [204,205]. Thus, they provide protection against several degenerative disorders, including brain dysfunction, cancer, and cardiovascular diseases [5]. Phenolic compounds reported from mushrooms include protocatechuic, p-hydroxybenzoic, p-coumaric, and cinnamic acids [204].

### 4.6. Vitamins

Vitamins are essential nutrients in the human body. They play an important role in bodily functions, such as metabolism, immunity, and digestion. Mushrooms are a good source of vitamins, especially of group B, namely thiamine (vitamin B1), riboflavin (vitamin B2), pyridoxine (vitamin B6), pantothenic acid (vitamin B5), nicotinic acid/niacin and its amide named nicotinamide (vitamin B3), folic acid (vitamin B9), and cobalamin (vitamin B12) [217,218]. Other vitamins, such as biotin (vitamin B8), tocopherol (vitamin E), and ergosterol, a precursor of vitamin D2, are also present [217]. Mushroom species such as Boletus edulis have a high group B content [219]. *Pleurotus ostreatus* contains high amounts of folacine (vitamin B9), and vitamins B1 and B3 [218]. *Lentinula edodes* and *Boletus edulis* have a high content of vitamin D [218]. The most common vitamin D in mushrooms is vitamin D2, which can also be found in vegetables and, thus, can be used as food supplements for vegetarians. Vitamin D4, 22-dihydroergocalciferol, can also be found in some mushrooms (agarics, morel, chanterelle) [220], but in small amounts [221]. Vitamin D has been suggested to have some therapeutic applications in the treatment of several diseases. In recent years, several clinical trials have been performed to investigate the therapeutic value of vitamin D in hyperproliferative diseases, secondary hyperparathyroidism, multiple sclerosis, rheumatoid arthritis, Crohn disease, type I diabetes, systemic lupus erythematosus, and various malignancies [222,223]. Vitamin D also helps in maintaining a healthy immune system by signaling the immune cells and promoting their ability to metabolize 25(OH)D3 into its active form 1,25(OH)2D3 [224].

### 4.7. Other Bioactive Compounds

Agaritine is an aromatic hydrazine-derivative compound identified in *Agaricus* species (Figure 2). It belongs to the IARC Group 3 carcinogens [225]. This compound is said to be toxic to animals and humans in large doses, but the review of Roupas et al. [225] stated that consumption of cultivated *A. bisporus* mushrooms poses no known toxicological risk to healthy humans. This compound could potentially be developed into antiviral drugs [226,227]. It also exhibits antitumor effects against leukemic cells *in vitro* [228].

Cordycepin, or 3’-deoxyadenosine, is the most vital bioactive compound produced by *Cordyceps* (Figure 2). The structure of cordycepin consists of a purine molecule attached to one ribose sugar moiety [229]. The bioactivities demonstrated by this compound are antiaging, antiarthritic, anticancer, antidiabetic, antifungal, antihyperlipidemia, anti-inflammatory, antimalarial, anti-osteoporotic, antioxidant, antiviral, hepato-protective, hypo-sexuality, immunomodulatory, weight-regulating, and many more nutraceutical and pharmaceutical applications in cardiovascular diseases, as well as general applications for maintaining good health [229,230,231,232,233,234]. Cordycepin isolated from *Cordyceps militaris* significantly inhibited the growth of MCF-7 human breast cancer cells with an IC_50_ value of 9.58 µM [235].

Erinacines isolated from the cultured mycelia of *H. erinaceus* belong to the group of cyathin diterpenoids (erinacines A-K, P, and Q), which have been shown to have an enhancing effect on nerve growth factor synthesis in vitro [236,237]. Erinacines show biological activities, including acting as stimulators of nerve growth factor (NGF) synthesis, suggesting it could be useful as a treatment for neurodegenerative disorders and peripheral neuropathy [238]. Erinacine A-enriched *H. erinaceus* mycelial extract was previously demonstrated to have antidepressant like effects in mice, activating the BDNF/TrkB/PI3K/Akt/GSK-3β pathways and blocking NF-κB mediated signaling [239]. Erinacine A also inhibited the growth of DLD-1 colorectal cancer cells [240] (Figure 2). Three capsules of 350 mg/g erinacine A-enriched *H. erinaceus* (EAHE) treatment for 49 weeks demonstrated higher positive results in patients with mild AD and achieved a better contrast sensitivity compared to the placebo group, suggesting that EAHE is safe, well tolerated, and may be important in achieving neurocognitive benefits [241].

Ganoderic acids are triterpenoids used as adjuvants in therapies and other medications. They can be used to treat hepatitis, fatigue syndrome, and prostate cancer [242]. Ganoderic acid also showed neuroprotective effects against the STZ-induced type I diabetes mellitus mouse model, with prebiotic effects on the gut microbiota allowing the growth of diabetes resistant bacteria [243]. It also worked as an antioxidant, exhibiting downregulation in Notch-1 mRNA expression and inhibiting proliferation, viability, and ROS activity in IMR-32 cells, thus can be potentially used to treat neuroblastomas [244].

Hericenones are also isolated from *Hericium erinaceus*. Just like erinacines, hericecones also promote NGF synthesis, wherein hericenone D (Figure 2) has almost the same degree of activity as the potent stimulator epinephrine [238]. They have been found to have anti-obesity properties which decrease fat cell number and improve body fat condition [245]. They also have strong antiplatelet activity [246]. It is important to note that hericenones have only been reported in the fruiting bodies of *H. erinaceus*, while erinacines have been found only in mycelium [238].

Lovastatin is a lactone metabolite isolated from the fungus *Aspergillus terreus* and also found in some mushrooms [247,248] (Figure 2). It has cholesterol-lowering, potential antineoplastic, and antitumor activities [248,249,250]. In comparison with other adjuvants, lovastatin showed more efficacious results than gemfibrozil in the reduction of total cholesterol (23% v 9%, P < 0.001) and low-density lipoprotein (LDL) cholesterol (28% v 2%, P < 0.001) [251].

## 5. Macrofungi Nutraceutical Market Overview

**Figure 3:**
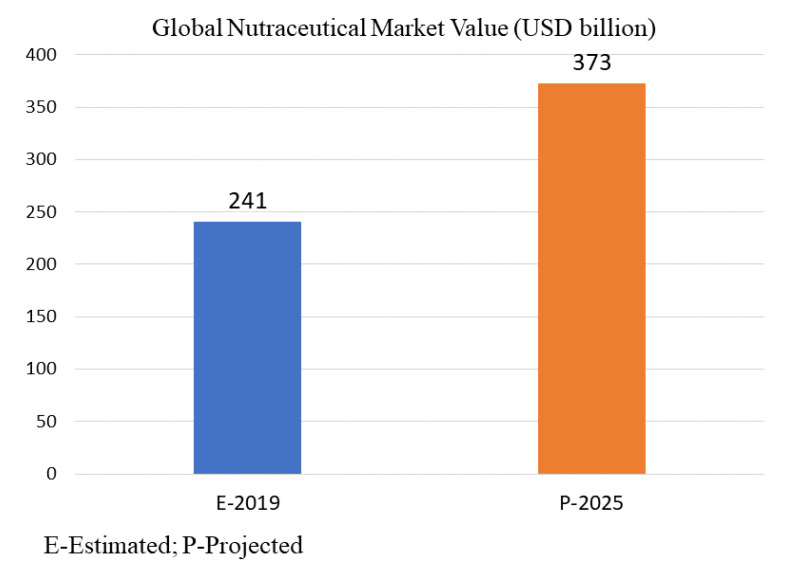
Global market value of nutraceuticals estimated in 2019 and projected in 2025 by PMMI Business Intelligence [253].

In the last decade, nutraceuticals and dietary supplements have increased in market value, owing to their role in the prevention of health risks and improving the health quality of human beings. The industry of nutraceuticals first emerged in the 1990s [252]. The nutraceutical market became more competitive when many major pharmaceutical and food companies ventured into the nutraceutical arena. According to the study conducted by PMMI Business Intelligence [253], the global nutraceutical market was worth approximately USD 241 billion in the year 2019, and is expected to bloom up to USD 373 billion by 2025 (Figure 3). The projection is that it will therefore grow at a 7.5% compound annual growth rate (CAGR) [254].

**Figure 4:**
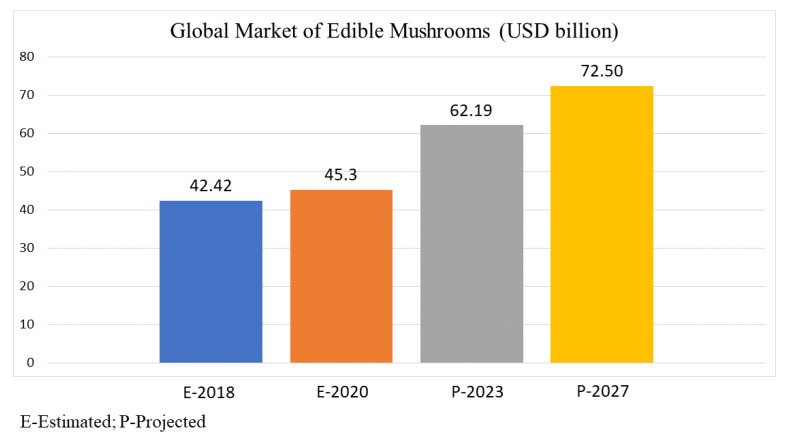
Global annual market revenue (USD billion) of edible mushrooms estimated and projected by Research and Markets [13].

Medicinal mushrooms are joining the nutraceutical market. The health-promoting benefits of consuming mushrooms have been a driving force in their increased market value. The global edible mushrooms market is forecasted to grow at a high CAGR of 7.9% during 2020–2027 [255]. Edible mushrooms had a global market value of USD 42.42 billion in the year 2018, USD 45.3 billion in 2020, and are forecasted to increase up to USD 62.19 billion in 2023, and USD 72.5 billion by 2027, growing at a CAGR of 7% over the analysis period 2020–2027 [13] (Figure 4). According to Market Data Forecast [256], the market value of edible mushrooms for Europe in 2018 was USD 13.71 billion, while Asia Pacific had a market value around USD 12.79 billion. It is expected to rise to USD 21.67 billion for Europe and USD 20.18 billion for Asia Pacific in 2023.

The market report of Technavio [10] projected that the medicinal mushroom market size will increase by USD 13.88 billion from 2018–2022 based on the analysis of the market-based products of Reishi mushroom, Chaga mushroom, and other medicinal mushrooms in the Americas, Asia-Pacific (APAC), Europe, and the Middle East and Africa (EMEA). Moreover, the growth of the population of people adopting a vegan diet will also likely result in an increase of the medicinal mushroom market CAGR to over 9% during 2018–2022 [10]. Mushroom nutraceuticals are often prepared as dietary supplements. The global dietary supplements market size is estimated to be valued at USD 136.2 billion in 2020 and projected to reach USD 204.7 billion by 2026, with a CAGR of 7.0% during the forecast period [257]. Changes in lifestyle, dietary habits, and positive outlook towards sport nutrition are the major factors driving the demand for dietary supplements.

Several pharmaceuticals (coriolan, krestin, lentinan, schizophyllan, etc.) formulated from medicinal mushrooms are already available in the world market [258]. The market value of glucans is more than USD 200 million, and expected to grow rapidly in medical, food, and cosmetic industries [259]. The global *Ophiocordyceps sinensis* (as *Cordyceps sinensis*) and *Cordyceps militaris* extract (cordycepins) market size was valued at USD 473.4 million in 2018, with a CAGR of 10.4% during the forecast period (2018–2026), and is predicted to surpass USD 1 billion by 2026 [260]. Asia Pacific (mainly China) is considered as the main producer of cordycepin from *O. sinensis* and *C. militaris* [229].

The market for lentinan is also expected to rise, with the global market valued at USD 10 million in 2020. It is predicted it will reach USD 12 million by the end of 2027, growing at a CAGR of 3.3% during the forecast period 2021–2027 [261].

Lovastatin and other statins are currently witnessing a stable market growth. According to Research and Markets [262], the increasing incidence of hypercholesterolemia among the geriatric population worldwide is among the key factors driving the growth of the market. The increasing consumer preference for more affordable over-the-counter (OTC) drugs is also another factor contributing to the market growth of statins.

## 6. Future Perspective

Nutraceuticals are of great importance for maintaining human health; thus, the market value for this industry is surging. Macrofungi produce many important bioactive compounds, with polysaccharides as the most promising nutraceuticals, having anticancer, antibacterial, antidiabetic, antiviral, immunoregulatory, and immunostimulatory properties, as well as hepato- and kidney-protective effects, antiaging activity, and many more bioactivities. Moreover, bioactive proteins, fats, and vitamins also present promising nutraceutical applications.

The growing demand for functional food and dietary supplements will cause a surge in product development and innovation, resulting in the increased market value of nutraceuticals, as projected by some research market studies. The diverse and unique marketing strategies by key players will also enable the health industry to grow in market value in the coming years [263]. Moreover, investment in research and development (R&D) to verify and confirm health claims, find innovative approaches, and market research are the driving forces for the success of the nutraceutical industry.

The consumption of medicinal mushrooms as a component of functional food and dietary supplementation is expected to have a remarkable upsurge in the future. The converging trends and popularity of eastern herbal medicines, natural/organic food product preference, gut-healthy products, and the positive outlook towards sport nutrition are supporting the growth in the medicinal mushroom market. The major drawback for medicinal mushrooms would be the taste, in which the medicinal mushroom industry spends extra cost in order to mask the bitter or mud-like tastes [264].

All the medicinal claims relating to medicinal mushrooms should be backed up by scientific studies, since the trust and confidence of customers are greatly influenced by the opinion of scientific communities. The popularity of medicinal mushrooms is directly related to research and development. The key strategies to success in mushroom nutraceuticals are consumer awareness, innovation, and educational marketing. According to Persistence Market Research [264], these products can get high-end consumers by marketing them through fashion magazines, spas, and other beauty supplement stores. There is also an expected rise of market value in developing countries where retail stores are stacked with functional food and beverages. It was also suggested that collaborations with various herbal supplement distributing chains would further increase the growth of mushroom nutraceuticals.

Only a handful of mushrooms have been considered economically important as nutraceuticals, namely *Agaricus bisporus* (button mushroom), *Ganoderma lingzhi*/*G. sichuanense* (Reishi), *Grifola frondosa* (maitake), *Hericium erinaceus* (lion’s mane), *Lentinula edodes* (shiitake), *Ophiocordyceps sinensis* (cordyceps), and *Trametes versicolor* (turkey tail). However, many other medicinal mushrooms are very promising, such as *Auricularia auricular-judae, Calocybe indica*, *Pleurotus* spp., *Schizophyllum commune*, and *Volvariella volvacea.* Opportunities for exploring mushrooms are enormous, but proper identification is very important, and further studies on the toxicity are necessary since numerous mushrooms are toxic and can be fatal when consumed.

The bioactive compounds from macrofungi hold a promise for future innovations for drug development, and as supplements to combat and prevent human diseases. However, the high price and low public awareness of medicinal mushrooms have become market limitations. In addition, the lack of quality control is also a major area of concern for mushroom nutraceuticals. Further research regarding the nutraceutical applications of macrofungi should be conducted to further validate their efficacy and safety.

## Figures and Tables

**Table 1:** Nutrient content of some economically important and other selected mushroom species.

Species	Moisture (g/100 g FW or DW)	Ash (g/100 g DW)	Proteins (g/100 g DW)	Fat (g/100 g DW)	Carbohydrates (g/100 g DW)	References
*Agaricus bisporus*	90.09 ± 0.07 (FW)	9.17 ± 0.52	24.43 ± 0.10	3.06 ± 0.03	53.10 ± 0.56	[38]
*Agaricus brasiliensis*	6.5 ± 0.11 (DW)	8.9 ± 0.09	37.3 ±0.22	9 2.4 ± 0.03	44.9 ± 2.5	[39]
*Agaricus campestris*	88.17 ± 0.44 (FW)	23.16 ± 0.00	18.57 ± 0.00	0.11 ± 0.00	58.16 ± 0.00	[40]
*Agaricus comtulus*	87.94 ± 0.77 (FW)	28.14 ± 0.18	21.29 ± 0.83	0.46 ± 0.00	50.11 ± 0.89	[40]
*Amanita battarrae* (as *A. umbrinolutea*)	73.60 ± 0.17 (FW)	28.86 ± 0.00	16.78 ± 0.00	6.77 ± 0.00	47.59 ± 0.00	[40]
*Amanita caesarea*	*-*	6.05 ± 0.01	34.77 ± 0.06	3.50 ± 0.00	55.63 ± 0.06	[41]
*Armillaria mellea*	88.27 ± 0.60 (FW)	6.78 ± 1.28	16.38 ± 1.34	5.56 ± 0.53	71.28 ± 1.06	[42]
*Armillaria mellea*	*-*	7.95 ± 0.02	24.47 ± 0.12	2.10 ± 0.02	65.47 ± 0.15	[41]
*Armillaria tabescens*	*-*	7.63 ± 0.15	22.90 ± 0.20	2.54 ± 0.03	66.87 ± 0.06	[41]
*Auricularia auricula-judae*	88.9 ± 0.02 (FW)	3.15 ± 0.3	56.92 ± 0.01	-	18.67 ± 0.01	[43]
*Auricularia nigricans* (as *A. polytricha*)	82.01 ± 0.04 (FW)	8.44 ± 0.8	42 ± 0.02	-	16.03 ± 0.02	[43]
*Auricularia thailandica*	80.75 ± 0.20 (FW)	4.30 ± 0.02	12.99 ± 0.05	2.93 ± 0.66	-	[44]
*Boletus aereus*	91.65 ± 1.04 (FW)	8.87 ± 0.10	17.86 ± 0.96	0.44 ± 0.08	72.83 ± 0.90	[45]
*Boletus aureus*	*-*	6.25 ± 0.02	27.17 ± 0.15	4.47 ± 0.02	62.10 ± 0.10	[41]
*Boletus edulis*	89.15 ± 0.90 (FW)	5.53 ± 0.23	21.07 ± 0.66	2.45 ± 0.09	70.96 ± 0.66	[45]
*Boletus fragrans*	77.99 ± 0.07 (FW)	4.74 ± 0.19	17.15 ± 0.04	1.83 ± 0.17	76.29 ± 0.27	[46]
*Boletus reticulatus*	91.10 ± 2.21 (FW)	19.72 ± 0.25	22.57 ± 2.08	2.55 ± 0.01	55.16 ± 2.03	[45]
*Bovista aestivalis*	73.23 ± 0.93 (FW)	31.86 ± 0.20	15.59 ± 1.23	0.18 ± 0.02	52.37 ± 1.31	[40]
*Bovista nigrescens*	76.41 ± 0.18 (FW)	3.24 ± 0.17	20.94 ± 0.31	3.64 ± 0.96	72.18 ± 0.76	[40]
*Bovistella utriformis* (as *Calvatia utriformis*)	78.00 ± 1.36 (FW)	17.81 ± 0.22	20.37 ± 0.49	1.90 ± 0.01	59.91 ± 0.40	[46]
*Calocybe gambosa*	90.92 ± 1.08 (FW)	13.89 ± 1.41	15.46 ± 0.24	0.83 ± 0.11	69.83 ± 1.22	[42]
*Cantharellus cibarius*	*-*	9.44 ± 0.01	21.57 ± 0.21	2.88 ± 0.02	66.07 ± 0.23	[41]
*Chlorophyllum rhacodes*	88.28 ± 0.33 (FW)	12.10 ± 0.31	19.32 ± 0.04	3.29 ± 0.33	65.29 ± 0.48	[40]
*Clavariadelphus pistillaris*	84.22 ± 1.78 (FW)	20.77 ± 0.86	16.27 ± 0.24	0.59 ± 0.07	62.37 ± 0.48	[40]
*Clavariadelphus truncatus*	90.97 ± 1.29 (FW)	12.86 ± 0.33	15.98 ± 0.15	1.54 ± 0.25	69.62 ± 0.37	[40]
*Clitocybe costata*	76.92 ± 2.11 (FW)	10.87 ± 1.36	17.27 ± 0.25	1.50 ± 0.00	70.36 ± 1.10	[40]
*Clitocybe odora*	88.49 ± 3.03 (FW)	9.55 ± 0.68	17.33 ± 1.37	2.46 ± 0.04	70.66 ± 1.09	[42]
*Clitopilus prunulus*	89.78 ± 1.46 (FW)	30.19 ± 2.50	18.13 ± 0.37	1.01 ± 0.06	50.66 ± 2.21	[46]
*Coprinus comatus*	85.19 ± 0.50 (FW)	12.85 ± 0.42	15.67 ± 0.23	1.13 ± 0.05	70.36 ± 0.26	[42]
*Coprinus comatus*	4.2 ± 0.06 (DW)	13.2 ± 0.42	22.7 ± 0.37	1.3 ± 0.02	58.6 ± 5.1	[39]
*Cordyceps militaris*	7.7 ± 0.61 (DW)	5.4 ± 0.16	29.7 ± 0.42	2.9 ± 0.18	54.3 ± 5.5	[39]
*Fistulina hepatica*	*-*	8.2 ± 0.10	22.6 ± 0.20	3.17 ± 0.02	66.0 ± 0.10	[41]
*Flammulina velutipes*	5.0 ± 0.13 (DW)	8.3 ± 0.08	23.4 ± 0.19	2.1 ± 0.10	61.2 ± 4.3	[39]
*Ganoderma lingzhi/G. sichuanense* (as *Ganoderma lucidum*)	5.1 ± 0.16 (DW)	1.0 ± 0.00	9.2 ± 0.32	1.1 ± 0.01	83.6 ± 4.4	[39]
*Grifola frondosa*	4.8 ± 0.08 (DW)	4.7 ± 0.07	18.3 ± 0.34	5.3 ± 0.09	66.9 ± 8.4	[39]
*Hemileccinum impolitum* (as *Boletus impolitus*)	88.90 ± 1.45 (FW)	24.43 ± 0.84	16.01 ± 0.02	2.94 ± 0.33	56.63 ± 0.84	[40]
*Hericium erinaceus*	6.2 ± 0.14 (DW)	6.8 ± 0.22	20.8 ± 0.43	5.1 ± 0.11	61.1 ± 3.6	[39]
*Hortiboletus engelii* (as *Boletus armeniacus*)	71.50 ± 0.43 (FW)	12.09 ± 0.35	18.25 ± 0.06	1.56 ± 0.42	68.10 ± 0.51	[40]
*Hygrophorus chrysodon*	92.09 ± 1.01 (FW)	26.91 ± 1.99	15.11 ± 0.18	3.48 ± 0.09	54.51 ± 1.28	[40]
*Hygrophorus pustulatus*	93.03 ± 0.79 (FW)	14.04 ± 0.14	18.64 ± 0.40	3.06 ± 0.51	64.26 ± 0.72	[46]
*Hygrophorus russula*	*-*	8.18 ± 0.02	32.47 ± 0.06	6.00 ± 0.10	53.33 ± 0.06	[41]
*Infundibulicybe gibba* (as *Clitocybe gibba*)	72.66 ± 0.99 (FW)	20.68 ± 0.15	14.59 ± 0.27	4.29 ± 0.00	60.45 ± 0.23	[40]
*Lactifluus piperatus*	80.03 ± 0.02 (FW)	5.38 ± 0.6	19.33 ± 0.02	-	9.2 ± 0.07	[43]
*Laetiporus sulphureus*	49.8 ± 0.02 (FW)	4.81 ± 0.5	22.73 ± 0.01	-	7.65 ± 0.01	[43]
*Lentinula edodes*	7.3 ± 0.10 (DW)	5.1 ± 0.05	18.5 ± 0.16	0.8 ± 0.01	68.3 ± 4.7	[39]
*Lentinula edodes*	82.8 ± 0.01 (FW)	5.59 ± 0.3	43.81 ± 0.02	-	38.44 ± 0.01	[43]
*Lentinus sajor-caju*	85.1 ± 0.02 (FW)	8.41 ± 0.2	62.27 ± 0.02	-	6.81 ± 0.01	[43]
*Lentinus sajor-caju* (as *Pleurotus sajor-caju*)	89.58 ± 0.19 (FW)	7.46 ± 0.30	25.65 ± 0.05	1.96 ± 0.12	52.46 ± 0.43	[38]
*Lentinus squarrosulus*	87.3 ± 0.02 (FW)	10.66 ± 0.4	27.86 ± 0.01	-	9.32 ± 0.01	[43]
*Lentinus squarrosulus* var. *squarrosulus*	86.2 ± 0.01 (FW)	3.12 ± 0.2	18.77 ± 0.02	-	19.14 ± 0.01	[43]
*Lentinus tigrinus*	73.7 ± 0.04 (FW)	3.41 ± 0.2	31.85 ± 0.03	-	16.09 ± 0.3	[43]
*Lepista nuda*	*-*	6.03 ± 0.02	34.37 ± 0.15	3.23 ± 0.01	56.33 ± 0.15	[41]
*Leucoagaricus leucothites*	85.29 ± 1.00 (FW)	26.46 ± 0.01	20.51 ± 0.47	1.10 ± 0.15	51.93 ± 0.53	[40]
*Lycoperdon echinatum*	85.24 ± 0.48 (FW)	9.43± 0.23	23.52 ± 2.20	1.22 ± 0.20	65.83 ± 2.09	[46]
*Lycoperdon umbrinum*	71.98 ± 0.32 (FW)	33.14 ± 1.06	14.53 ± 0.07	0.37 ± 0.00	51.96 ± 0.70	[40]
*Lyophyllum decastes*	87.38 ± 1.40 (FW)	7.38 ± 0.64	25.52 ± 3.49	2.10± 0.12	64.99 ± 2.96	[46]
*Macrolepiota excoriata*	88.92 ± 1.57 (FW)	28.98 ± 1.11	25.28 ± 2.64	1.55 ± 0.10	44.19 ± 2.14	[46]
*Morchella esculenta* (as *Morchella conica)*	*-*	14.6 ± 0.30	7.5 ± 0.40	2.8 ± 0.10	75.0 ± 0.40	[47]
*Neoboletus erythropus* (as *Boletus erythropus)*	88.36 ± 1.49 (FW)	25.90 ± 0.28	20.92 ± 0.05	0.75 ± 0.02	52.44 ± 0.20	[46]
*Pleurotus ostreatus*	8.2 ± 0.07 (DW)	7.1 ± 0.06	33.5 ± 0.22	2.3 ± 0.07	48.9 ± 2.7	[39]
*Ramaria aurea*	88.52 ± 0.12 (FW)	5.68 ± 0.74	14.60 ± 0.10	2.26 ± 0.05	77.47 ± 0.61	[40]
*Ramaria largentii*	*-*	6.67 ± 0.12	28.80 ± 0.46	5.67 ± 0.12	58.87 ± 0.25	[41]
*Russula cyanoxantha*	85.44 ± 0.99 (FW)	7.03 ± 0.87	16.80± 0.06	1.52 ± 0.52	74.65 ±1.01	[46]
*Russula delica*	*-*	5.61 ± 0.03	26.10 ± 0.30	4.44 ± 0.04	63.87 ± 0.31	[41]
*Russula olivacea*	84.58 ± 1.01 (FW)	37.78 ± 5.20	16.84 ± 0.05	1.99 ± 0.44	43.38 ± 3.71	[46]
*Schizophyllum commune*	69.8 ± 0.02 (FW)	6.02 ± 0.6	24.42 ± 0.02	-	5.31 ± 0.01	[43]
*Suillus variegatus*	90.77 ± 0.76 (FW)	15.36 ± 2.10	17.57 ± 0.56	3.31 ± 0.49	63.76 ± 2.17	[40]
*Termitomyces heimii*	81.1 ± 0.02 (FW)	5.66 ± 0.02	60.53 ± 0.01	-	22.74 ± 0.01	[43]
*Tremella fuciformis*	5.5 ± 0.18 (DW)	6.5 ± 0.14	13.0 ± 0.12	2.1 ± 0.08	72.9 ± 6.4	[39]

FW, fresh weight; DW, dry weight; (*-*) No data available.

**Table 2:** Experimental in vivo and in vitro studies over the last 5 years (2016–2021) of bioactive polysaccharides from economically important macrofungi.

Mushroom Species	Name of Fraction(s)	Bioactivity	Target Cells/Experimental Subjects	References
*Agaricus bisporus*	*Agaricus bisporus* neutral polysaccharides (Abnp1001 and Abnp1002) and *Agaricus bisporus* all polysaccharides (Abap1001, and Abap1002)	Hepato-protective activity	CCl_4_-induced hepatic injury in mice	[7]
	AlAPS and their three purified fractions (AlAPS-1, AlAPS-2, and AlAPS-3)	Antiaging, antioxidant, and hepatoprotective effects, prevent age-related diseases	Fresh liver and blood samples of male Kunming strain mice	[66]
	Mannogalactoglucan polysaccharide	Antitumor activity (lung cancer)	Human hepatocarcinoma cells (HepG2)	[67]
	AcAPS and its major purified fractions (AcAPS-1, AcAPS-2 and AcAPS-3)	Antiaging and antioxidant effects	Fresh liver and kidney samples of male Kunming strain mice	[68]
	*Agaricus bisporus* fruiting body polysaccharide (FPS)	Hepato-protective activity	CCl_4_-induced liver injury in mice	[69]
	Glucogalactomanan polysaccharide TJ3	Immunostimulatory activity	RAW 264.7 cells	[70]
*Ganoderma lingzhi/G. sichuanense* (as *Ganoderma lucidum*)	*Ganoderma lucidum* polysaccharides (GLP)	Immunomodulatory effect	Mice immunized with GLPL/OVA	[71]
	GLP	Antitumor activity (colorectal cancer)	Colorectal cancer HT29 (p53R273H) and SW480 (p53R273H&P309S) cells	[72]
	GLP	Neuroprotective effects	Rat cerebellar granule cells (CGCs)	[73]
	GLP	Anticancer activity (prostate cancer)	Human prostate cancer cells LNCaP	[74]
	GLP	Antitumor (brain glioma) and immunomodulatory activities	Glioma-bearing rats	[75]
	GLP	Hypoglycemic effect	Type 2 diabetes mellitus (T2DM) rats’ blood liver and skeletal muscles	[76]
	Degraded *Ganoderma lucidum* polysaccharides (GLP_UD_)	Hypolipidemic and antioxidant activities	Blood, heart, spleen, liver and kidney of male Kunming mice	[77]
	GLP	Antidiabetic activity	T2DM rats’ blood	[78]
*Grifola frondosa*	*Grifola frondosa* polysaccharides (GFP)	Anticancer activity (breast cancer)	MCF-7 and MDA-MB-231 cells, as well as in nude mice bearing MCF-7 tumor xenografts.	[79]
	GFP	Memory enhancement and antiaging activities	20-month-old rats	[80]
	GFP-N	Hypoglycemic and prebiotic activities	Diabetic mouse livers	[81]
	GFP	Hypoglycemic and hypolipidemic activities	Diabetic mice induced by HFD and streptozotocin (STZ)	[82]
*Hericium erinaceus*	Hydroxyethylated derivative of HEP	Immunomodulatory activities	RAW264.7 macrophages	[83]
	Selenium derivatives (sHEPs)	Immunostimulant activity	Dendritic cells	[84]
	*Hericium erinaceus* crude polysaccharide (HECP) and *Hericium erinaceus* refined polysaccharide (HERP)	Gastroprotective activity	Sprague–Dawley rats’ stomach	[85]
	Novel *Hericium erinaceus* polysaccharide HEP_N_	Gastroprotective activity	Human gastric epithelium (GES-1) cells	[86]
	*Hericium erinaceus* fruiting body polysaccharide (HEFP)-2b	Anticancer activity (colon cancer)	Colon cancer cells (HCT-116)	[87]
	Enzymatic hydrolysis of *Hericium erinaceus* polysaccharide (EHEP)	Immune-enhancement activity	Female Balb/c mice	[88]
*Lentinula edodes*	Mannogalactoglucan-type polysaccharides (WPLE-N-2 and WPLE-A0.5-2)	Anticancer and immunomodulating activities	Sarcoma 180-bearing mice	[64]
	Myeloid-derived suppressor cells	Immunosuppressive effects	Immortalized myeloid immune suppressor cell line (MSC2)	[89]
	*Lentinula edodes* polysaccharide (LEP)1	Antitumor activity	Human cervical carcinoma HeLa cells	[90]
	Residue polysaccharide (RPS) and its enzymatic-RPS (ERPS)	Antioxidant and anti-inflammatory activities	LPS-induced sepsis in mice	[91]
	LEP	Anticancer (colon cancer)	HT-29 colon cancer cells	[85]
	Acidic spent mushroom compost polysaccharides (ASMCP)	Antioxidant, anti-inflammatory and renoprotective effects	LPS-induced KI in mice	[92]
	Polysaccharide fractions (F1, F2 and F3)	Immunomodulatory effects	Female BALB/c mice	[93]
*Ophiocordyceps sinensis* (as *Cordyceps sinensis*)	*Cordyceps sinensis* polysaccharide (CSP1-2)	Antihypertensive effect	Spontaneously hypertensive rats (SHR)	[94]
	CPS-A	Protective effect	L02 cells	[95]
	CSP	Prebiotics	Cyclophosphamide (Cy)-induced intestinal mucosal immunosuppression and microbial dysbiosis in mice	[96,97]
	CSP	Anti-obesity	High-fat diet (HFD)-feeding C57BL/6J mice	[98]
	Docetaxel-loaded acetic acid conjugated *Cordyceps sinensis* polysaccharide (DTX-AA-CSP)	Drug carrier and anticancer (liver and colon cancers)	Human umbilical vein endothelial cells; human liver HepG2; colon cancer cells SW480	[99]
	CSP	Anticancer activity (colon cancer)	Colon cancer cell line HCT116	[100]
*Pleurotus eryngii*	*Pleurotus eryngii* polysaccharides PEP-1 and PEP-2	Antitumor	Human hepatoblastoma HepG-2 cells	[101]
	*Pleurotus eryngii* polysaccharide (PEP)	Hypolipidemic and hypoglycemic activities	KK-A^y^ mice	[102]
	water-soluble polysaccharide EPA-1	Immunoregulatory activity	RAW 264.7 cells	[103]
	PEP	Hypolipidemic effect	Mice with hyperlipidemia	[104]
	PEP	Neuroprotective effect	β-amyloid-induced neurotoxicity in cultured rat pheochromocytoma (PC12) cells	[105]
*Pleurotus ostreatus*	*Pleurotus ostreatus* polysaccharide (POP)	Regulating dyslipidemia effect	STZ-induced diabetic rats	[106]
	POP	Anticancer activity	Sarcoma 180 tumor cells	[107]
	POP	Regulating dyslipidemia effect	Fat-emulsion-induced hyperlipidemia rats	[108]
	POP	Anticancer (lymphoid cancer)	Murine lymphoid cancer cell line	[109]
	Selenium polysaccharide fraction (Se-POP-3)	Antitumor activity	Human cancer cell lines HepG2, MCF-7, SKOV3, HeLa, and PC-3	[110]
	Phosphorylated *Pleurotus ostreatus* polysaccharide (PPOP)	Hepatoprotective effect	Carbon tetrachloride-induced liver injury in mice	[111]
*Trametes versicolor*	Polysaccharopeptides PSPs-EH80	Antioxidative effect	HaCaT cells	[112]
	*Trametes versicolor* polysaccharide (TVP)	Anti-proliferative and anti-invasive effects	LoVo and HT-29 human colon cancer cells	[113]
	Intracellular polysaccharide extract of *Trametes versicolor* (IPTV) and extracellular polysaccharide extracts of *T. versicolor* (EPTV)	Antihyperlipidemic effects	HFD-induced hyperlipidemic mice	[114]

## Data Availability

Not applicable.
